# Ferulated Arabinoxylans and Their Gels: Functional Properties and Potential Application as Antioxidant and Anticancer Agent

**DOI:** 10.1155/2018/2314759

**Published:** 2018-08-16

**Authors:** Mayra Alejandra Mendez-Encinas, Elizabeth Carvajal-Millan, Agustín Rascon-Chu, Humberto Francisco Astiazaran-Garcia, Dora Edith Valencia-Rivera

**Affiliations:** ^1^Biopolymers, Research Center for Food and Development, CIAD, A.C. Carretera a La Victoria Km. 0.6, 83304 Hermosillo, SON, Mexico; ^2^Biotechnology, Research Center for Food and Development, CIAD, A.C. Carretera a La Victoria Km. 0.6, 83304 Hermosillo, SON, Mexico; ^3^Nutrition, Research Center for Food and Development, CIAD, A.C. Carretera a La Victoria Km. 0.6, 83304 Hermosillo, SON, Mexico; ^4^Department of Chemical Biological and Agropecuary Sciences, University of Sonora, Avenida Universidad e Irigoyen, 83621 Caborca, SON, Mexico

## Abstract

In the last years, biomedical research has focused its efforts in the development of new oral delivery systems for the treatment of different diseases. Ferulated arabinoxylans are polysaccharides from cereals that have been gaining attention in the pharmaceutical field due to their prebiotic, antioxidant, and anticancer properties. The antioxidant and anticancer properties of these polysaccharides make them attractive compounds for the treatment of cancer, particularly colon cancer. In addition, ferulated arabinoxylans can form covalent gels through the cross-linking of their ferulic acids. Due to their particular characteristics, ferulated arabinoxylan gels represent an excellent alternative as colon-targeted drug delivery systems. The aim of the present work is to review the physicochemical and functional properties of ferulated arabinoxylans and their gels and to present the future perspectives for potential application as antioxidant and anticancer agents.

## 1. Introduction

Consumption of whole grains is associated with the prevention of cardiovascular diseases, diabetes, obesity, and cancer [1]. The dietary fiber and the antioxidant compounds of grains play an important role providing such benefits.

An inverse relationship between the dietary fiber of whole grain consumption and total cancer death has been established [2]. Particularly, a case study suggested that higher intake of dietary fiber reduces the risk of incident colorectal adenoma and distal colon cancer [3]. In addition, dietary fiber has shown to reduce type 2 diabetes mellitus risk through glycemic control or decrease energy intake, reduce blood glucose excursions, and lower insulin responses [4]. Moreover, insoluble antioxidants of whole grains which are bounded to arabinoxylan side chains can be released by microbial enzymatic hydrolysis in the colon and adsorbed, exhibiting an antioxidant protection [1]. On the other hand, certain components of dietary fiber such as arabinoxylans (AX) have prebiotic properties. In fact, phenolic acids of AX, such as ferulic acid (FA), exert antioxidant activity [5]. In this way, AX have gained attention in the pharmaceutical field due to their interesting functional and biological properties.

AX are nonstarch polysaccharides in the cell wall of cereal grains. A unique property of AX is their ability to form covalent gels by the oxidative coupling of the FA [6]. Due to their covalent nature, these gels have interesting characteristics such as high water absorption capacity and stability to pH, temperature, and ionic charges [7]. In addition, AX gels exhibit antioxidant activity [8] and can be fermented by the colonic microbiota [9–11]. Furthermore, several studies have demonstrated the biological properties of AX, particularly their prebiotic, antioxidant, and more recently anticancer properties [12–16]. The anticancer activity of AX has been largely related to their prebiotic and antioxidant properties [17–19]. Thus, the particular characteristics and functional properties of AX make them promising polysaccharides for biopharmaceutical purposes.

The prebiotic and antioxidant properties of AX depend on its structural characteristics. It has been established that the presence and appearance of FA in AX impacts directly in its antioxidant and prebiotic properties [20]. A previous study showed that highly feruloylated AX oligosaccharides (AXOS), hydrolytic degradation products of AX, were less fermented than AXOS depleted in FA [20]. This appears to be a great advantage due to the selective inhibition of the growth of certain nonbeneficial bacteria, but the growth of probiotic bacteria, such as *Lactobacillus* and *Bifidobacterium*, which are able to produce FA esterases to release FA from AXOS and AX [21, 22]. In addition, the presence and amount of FA result to be the principal factor in providing the antioxidant capacity to AX and AXOS as has been well documented previously [10, 20]. Then, it could be interesting to investigate how the cross-linking of AX could impact on the prebiotic and antioxidant properties of AX gels.

Recently, researchers have focused their attention on the development of novel bioactive materials as colon-targeted oral delivery systems for the treatment of diseases such as colon cancer [23, 24]. AX gels with anticancer activity could be potential candidates for use as matrices for drug delivery in the treatment of colon cancer. AX with a high content of FA lead to the formation of high cross-linked density gels [25]. Since AX exhibit anticancer activity, the effect of the oxidative gelation and the cross-linking density of the gels on such property need to be investigated. In this context, the objective of the present review focuses on the functional and biological properties of AX and their gels and their potential application as antioxidant and anticancer agents.

## 2. Arabinoxylans

### 2.1. Chemical Structure

**Figure 1:**
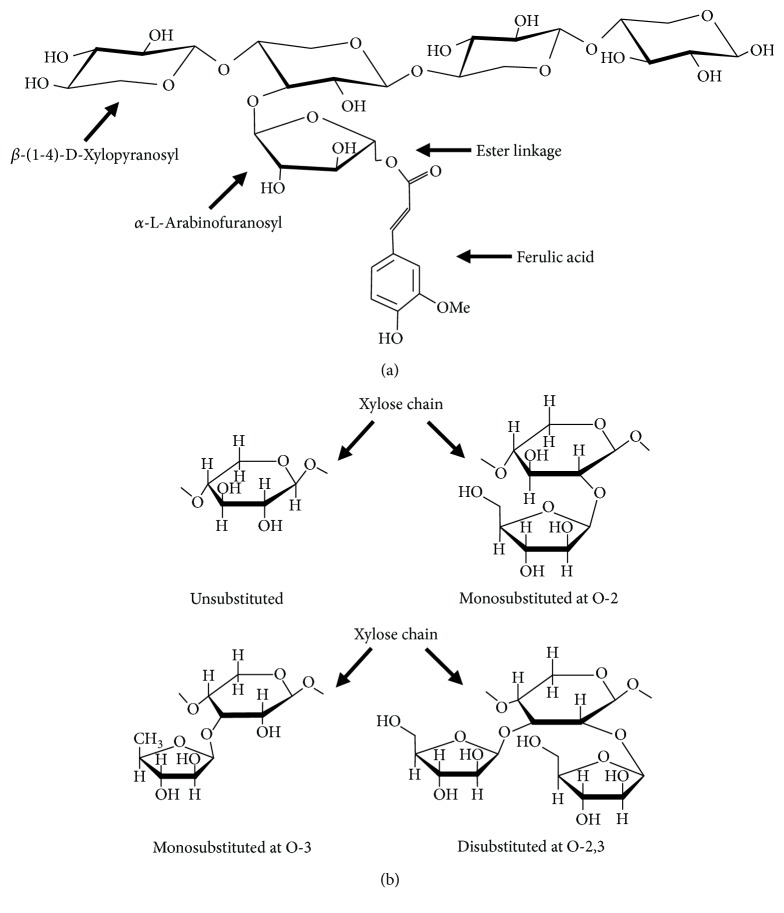
(a) Chemical structure of AX and (b) arabinose substitution in AX chain.

AX are polysaccharides from cereal grains constituted by a linear *β*-(1-4)-xylopyranosyl chain. Some *α*-L-arabinofuranosyl residues are linked to the main xylose chain at O-3 and/or O-2 positions, resulting in four different structures (monosubstituted at O-3 or O-2, disubstituted at O-2,3, and unsubstituted) (Figure 1(b)). The amount and distribution of these branches can vary depending on the source of the polysaccharide [26]. In addition to arabinose, some galactose, xylose, and glucuronic acid residues can exist as side branches in the main chain of AX [27].

A particular structural characteristic of AX is the presence of phenolic acids. Some FA and cumaric acid residues can be esterified to arabinose at the O-5 position [28] (Figure 1(a)). FA is the most abundant phenolic acid in AX, and its content depends on the origin of the tissue (Table 1). AX from endosperm contains very small amounts of FA, while AX from pericarp and aleurone layer are highly esterified to FA [20]. The FA contents in AX vary from 0.001 to 7.00 *μ*g/mg AX [29, 30]. High contents of FA (6–7.00 *μ*g/mg AX) have been detected in maize bran AX [25, 30], while AX extracted from finger millet bran and ispaghula seed contain very low or even undetectable amounts of FA (0.001 *μ*g/mg AX) [29, 31]. These differences could be related to the source of the polysaccharide as well as the method used for its extraction.

Usually, the structure of AX in different cereal tissues is similar, although some differences in the fine structure can drastically modify its functional properties. These differences are reflected in the degree of polymerization (DP), arabinoxylan to xylose ratio (A/X), amount and sequence of glycosidic bonds, and the presence of other substituents [7]. AX can be classified according to their solubility in aqueous solvents as water-extractable (WE-AX) and water-unextractable (WU-AX) AX. In cereals, the cross-linking between AX and other components from the cell wall form structures that are insoluble in water. Alkali treatments are used to hydrolyze such cross-links, allowing the release of AX chains from the cell wall and making them soluble in the aqueous environment [27].

The substitution degree in the AX structure can be determined by the A/X. The A/X may vary from 0.3 to 1.1 in AX from different cereals depending on the origin of the polysaccharide; AX from pericarp present higher A/X values than those for endosperm or aleurone layer [46, 47]. However, this parameter does not describe detailed and exhaustive structural characteristics of AX, and therefore, it cannot be used to characterize its fine structure.

The molecular weight (Mw) of AX can vary depending on the polysaccharide origin and the method used for its determination. The average Mw estimated for AX ranges from 10 to 10,000 kDa [7]. The Mw and the Mw distribution (polydispersity index (PI)) of AX can be affected by the extraction conditions (time, pH, and temperature) [48].

### 2.2. Physicochemical Characteristics

AX show physicochemical characteristics, such as solubility and viscosity, which provide them different functional properties. Similar to other polysaccharides, the water solubility of AX depends on certain parameters such as the chain-chain and chain-solvent interactions. In addition, some structural factors including the chain length and the presence and distribution of side groups can also modify the solubility of the polymers. The substitution pattern of the polysaccharide chain is the main parameter controlling the solubility of AX. Since the mechanism of aggregation in the AX is due to the intermolecular interactions of the unsubstituted regions of the polysaccharide chain, the presence of arabinose residues in the xylose chain is determinant for the solubility of AX [48].

AX form very high viscous solutions in aqueous environments. The apparent viscosity of the AX solutions is concentration- and shear rate-dependent. The viscosity values increase as the polymer concentration increases, and decrease as the shear rate increases [27]. The Mw is another important factor that determines the viscosity of the AX solutions. Izydorczyk and Biliaderis [49] demonstrated that wheat AX solutions with high Mw fractions showed weak elastic properties. The viscous behavior of AX solutions is the most important characteristic responsible for the functional properties that AX exhibit in the human gastrointestinal tract [27].

### 2.3. Functional Properties

Functional properties of AX such as prebiotic, antioxidant, and anticancer as well as its gelling capacity result to be of great interest for biomedical and pharmaceutical applications. Therefore, research has focused its efforts on exploiting different sources of AX in order to explore and take advantage of such properties for several applications (Table 1). Maize, wheat, and rice are the main sources of AX which have been investigated for biomedical and pharmaceutical applications. Maize and wheat AX gels have shown promising results for their potential application as controlled-release matrices [11, 32–35, 37]. On the other hand, the use of AX from rice bran has been widely investigated as an adjuvant in cancer immunotherapy [41, 42].

#### 2.3.1. Prebiotic

The products of AX degradation have been of great interest due to their prebiotic properties. Prebiotics are defined as “a non digestible compound that, through its metabolization by microorganisms in the gut, modulates the composition and/or activity of the gut microbiota, thus conferring a beneficial physiological effect on the host” [50]. AX are resistant to gastric acid, proteolytic enzymes, and absorption in the stomach or small intestine. In addition, AX are fermented by the gut microbiota and selectively stimulate the growth and/or activity of beneficial bacteria in the colon, so they may be considered as prebiotics.

AX stimulate the growth of beneficial bacteria for the gastrointestinal tract. *In vivo* assays have demonstrated that AX promote the growth of *Bifidobacterium* and *Lactobacillus*, both considered as beneficial species for the gut [51]. These modifications in the gut microbiota are associated with health benefits, reduction in gastrointestinal infections, and improvement in the mineral absorption and the suppression of colon cancer [52, 53].

It has been established that some phenolic acids modulate the composition of microbiota through the selective inhibition of some pathogenic bacteria, while the growth of commensal anaerobes and probiotic bacteria is less affected or even increased [22, 54]. In this regard, the growth of probiotic such as *Bifidobacterium* and *Lactobacillus* during AX fermentation can be explained by the fact that these bacteria produce the FA esterases to release the FA residues from AX [21, 55, 56].

Another benefit of AX as a prebiotic is the production of beneficial bacterial metabolites, such as short-chain fatty acids (SCFA). The fermentation of AX increases the production of acetic, propionic, and butyric acids. Particularly, AX are characterized by increasing the butyric acid levels, which play an important role in the maintenance of health and gastrointestinal function [57]. The fermentation of AX is associated with the growth of butyric acid-producing bacteria, such as *Eubacterium* and *Roseburia* [58]. Nielsen et al. [59] evaluated the effect of AX-rich and high-fat diets on the production of SCFA in pigs. The supplementation of the AX-rich diet increased the SCFA levels, particularly the butyric acid level which was 5-fold higher compared with that obtained for the high-fat diet.

Butyric acid is considered an essential metabolite for the human colon as it is the main source of energy for its epithelial cells (colonocytes), contributes to the maintenance of the gut barrier functions, and has immunomodulatory and anti-inflammatory properties [58]. In addition, the proliferation of butyrate-producing bacteria protects the colon from pathogenic microbiota [60]. For those reasons, AX have been considered as polysaccharides with excellent prebiotic properties. The consumption of this polysaccharide provides many health benefits, especially those related with the prevention of colon cancer.

Recently, long-chain AX (LC-AX) have demonstrated to modulate the luminal and mucosal microbiota. Experiments using a dynamic *in vitro* model of the human digestive tract (M-SHIME) showed that supplementation of LC-AX to the proximal colon compartments of the M-SHIME increased *Bifidobacterium* population in both lumen and mucus compared with the control. The levels of propionate as well as the activity of enzymes *β*-xylanase, *β*-xylosidase, and *α*-arabinofuranosidase were also increased in the lumen region. These findings suggest that LC-AX could exert a potential prebiotic effect on the host, as the mucosa-associated microbiota impacts directly in health by protecting against pathogen colonization and host immunity [61].

#### 2.3.2. Antioxidant

The antioxidant activity of AX has been mainly associated with their content of phenolic acids, particularly FA. Phenolic acids have beneficial effects against chronic and cardiovascular diseases, cancer, diabetes, inflammatory diseases, and aging [62]. Phenolic acids exhibit their antioxidant activity through diverse mechanisms such as free radical scavenging, metal chelation, and reducing potential, blocking the free radical chain, modulation of enzymatic activity, and alteration of signal transduction pathways [63–65].

The main function of antioxidants is delaying or prevention of the oxidation produced by free radicals [66]. The free radicals are generated by diverse factors such as normal metabolic activity, diet, and environment. In a natural manner, the body uses antioxidant endogenous enzymes as a defense mechanism against the free radicals. An increase in the production of free radicals and other reactive oxygen species exceeding normal levels in the body results in oxidative stress. This imbalance causes damage to biomolecules such as membrane lipids, lipoproteins, and DNA, increasing the risk of developing chronic diseases [67]. Therefore, the use of antioxidants results to be adequate to decrease the effects of free radicals and the risk of chronic diseases, such as cancer.

Although the antioxidant activity of AX is associated with the presence of phenolic acids, some studies suggest that this activity is mainly attributed to FA. The antioxidant activity of FA is attributed to its structural characteristics. The presence of electron-donating groups on its benzene ring gives it the property of terminating the free radical chain reactions. In addition, its COOH– group can bind to the lipid bilayer, providing protection against the free radicals attack and the lipid peroxidation [62]. Since FA is the most abundant phenolic acid in AX, it may be the main responsible for the antioxidant activity of the polysaccharide, as has been observed in different studies.

Recently, Kamboj and Rana [68] compared the antioxidant activity of maize bran gum with the antioxidant activity from other natural gums as xanthan and guar gums. They found that the maize fiber gum exhibited a higher antioxidant activity compared to the other gums, regardless of the method used for the determination. They suggested that maize fiber gum could be a promising excipient with antioxidant activity in the food and pharmaceutical industry.

Feruloyl oligosaccharides (FOS), hydrolytic products of AX, exhibit a protective effect on the cells against the damage produced by the free radicals. Wang et al. [14] evaluated the protective effect of FOS against the oxidative stress in rat plasma. The levels of oxidized glutathione and malondialdehyde and the activity of antioxidant enzymes in plasma from rats fed with the FOS diet decreased with respect to the control group. In a previous study, the protective activity of FOS against the oxidative DNA damage in normal human lymphocytes induced by hydrogen peroxide was investigated. The DNA damage was inhibited by FOS, observing a 91% inhibition of lymphocyte DNA damage at 500 *μ*mol/L as compared with control [69].

The content and appearance of FA determine the antioxidant capacity of AX. Higher contents of trimers of FA (tri-FA) in AX result in higher antioxidant activity [5]. This behavior is attributed to three units of FA, which provide higher amounts of OH– groups, increasing the hydrogen donor capacity and, therefore, protecting from radical scavenging [70]. This highlights the close relationship existing between the structural characteristics of the polysaccharide and its functional properties. Therefore, when discussing about the antioxidant capacity of AX, not only the FA content should be considered but also how it is found in the molecule. The knowledge of such structural characteristics would help to predict the antioxidant activity of AX in order to consider it for specific applications.

#### 2.3.3. Anticancer

Cancer is among the leading causes of death worldwide, and the second most common in the United States [71]. Usually, conventional cancer treatments including surgery, chemotherapy, and radiotherapy which focus on eliminating cancer cells are short-term effective, but not enough for a complete eradication of all cancer cells, resulting in recurrence of disease [72]. The repeated sessions of treatments lead to the suppression of the immune system and promote multidrug resistance and toxicity [41, 73]. Therefore, the search for natural products with chemopreventive properties and without side effects has been increasing.

AXOS exhibit protective effects against colon cancer, which have been related to their prebiotic effect. Femia et al. [17] observed that the administration of AXOS reduced the preneoplastic lesions in the colon of rats. The authors suggest that AXOS exhibited a chemopreventive effect on colon carcinogenesis due to their prebiotic activity. On the other hand, Glei et al. [74] showed that the fermentation products of wheat AX (SCFA) inhibited the growth of colon cancer cells (HT29) and induced the antioxidant activity of the endogenous enzyme glutathione transferase.

A close relationship between the proliferation of cancer cells and the antioxidant systems has been suggested. Cancer cells produce large amounts of hydrogen peroxide, which may favor mutations, damage, and invasion of other tissues. Then, cancer cell proliferation impacts directly in the antioxidant machinery, and according to this, some anticancer agents can act as antioxidants [19]. Thus, the antitumor potential of AX has been related to its antioxidant effect. Noaman et al. [19] supplied AX from rice to rats, which were previously inoculated with Erlich cancer cells. The results showed the inhibition of the development and growth of tumors, as well as a decrease in lipid peroxidation and increase in the activity of endogenous antioxidant enzymes (catalase, superoxide dismutase, and glutathione transferase). The authors suggested that AX exerted an antioxidant effect through its ability to increase the gene expression and activity of endogenous antioxidant enzymes in the cells and normalize the lipid peroxidation in blood, liver, and tumor tissue in animals bearing tumors.

In a previous study, the antitumor activity of MGN-3/Biobran on mice bearing a solid Erlich carcinoma (SEC) tumor was attributed to mechanisms involving induction of apoptosis and immune modulation. The administration of MGN-3 significantly decreased tumor volume (63.27%) and tumor weight (45.2%) in comparison to the control group. Flow cytometry and histopathological analyses showed an increase in the number of apoptotic SEC cells. In addition, an improvement of cytokine production was observed as shown by increasing levels of tumor necrosis factor-*α* and interferon-*γ*, while the levels of immune suppressing IL-10 were downregulated. In addition, the activity of natural killer (NK) cells was also increased [75].

The synergistic anticancer effect of MGN-3/Biobran with natural anticancer agents as well as chemotherapeutic drugs has been widely explored using *in vitro* studies. The synergistic apoptotic potential of MGN-3 and curcumin on human multiple myeloma cell line U266 was determined. Treatment of MGN-3 or curcumin alone showed an inhibition of cell proliferation in a dose-dependent manner. The combination of MGN-3 and curcumin caused a synergistic effect characterized by a decrease in cell number and an increase in apoptotic cells. The expression of the proapoptotic protein (Bax) increased, while antiapoptotic protein (Bcl-2) decreased, which favored apoptosis. These findings indicated that Biobran and curcumin synergize in the induction of apoptosis [76]. A similar behavior was observed when Biobran was combined with paclitaxel in order to sensitize human and murine breast cancer cells (MCF-7 and 4T1) to paclitaxel. A synergistic effect between Biobran and paclitaxel resulted in damage of DNA, enhancement of apoptosis, and inhibition of 4T1 cell proliferation [77].

In sum, researches have demonstrated the anticancer potential of the AX. Such property is attributed to both the antioxidant and prebiotic capacity of the polysaccharide. In addition, it is suggested that AX exerts its anticancer effect by a mechanism which involves its immune-modulation ability. *In vitro* as well as *in vivo* studies have evidenced the anticancer effect of AX on different types of cancer. Nevertheless, most studies highlight its beneficial effects in the prevention of colon cancer, due to the benefits of its prebiotic activity and immunostimulatory activity.

#### 2.3.4. Gelling

**Figure 2:**
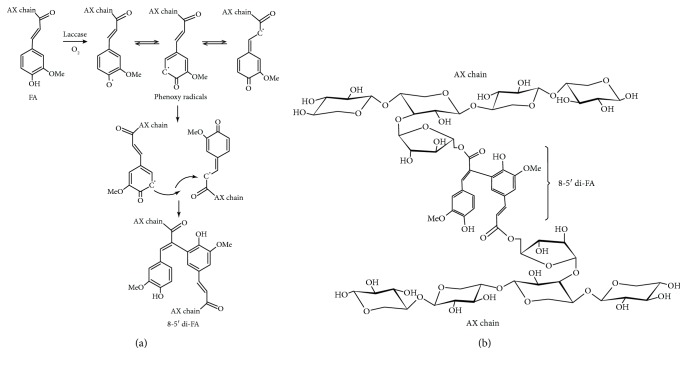
(a) Schematic representation of FA dimerization. (b) Covalent cross-linking of ferulated AX. Formation of 8-5′ di-FA is presented as an example. AX: arabinoxylan; FA: ferulic acid; di-FA: ferulic acid dimer.

A particular property of AX is their capacity to form covalent hydrogels. AX can gel through the covalent cross-linking of FA [6], under the action of different chemical (ferric chloride, ammonium persulphate) or enzymatic (laccase/O_2_, peroxidase/H_2_O_2_, linoleic acid/lipoxygenase) oxidizing agents [78–83] (Figure 2(b)). The ability of AX to gel depends on the concentration of the polysaccharide, Mw, and particularly the FA content [27].

The oxidative gelation of AX results from the dimerization of FA residues of adjacent polysaccharide chains leading to the formation of the three-dimensional network where the aqueous phase is retained. The FA dimerization mechanism occurs as follows: first, an oxidizing agent attacks the H atom of the OH group at the ring position of FA resulting in a phenoxy radical. Then, this radical is stabilized by resonance and located at three different positions onto the whole molecule, two on the aromatic ring (C-4 and C-5) and one at the double bound (C-8) of its side chain. In the next step, the cross-linking between two phenoxy radicals is carried out; the coupling of unpaired electrons of two different radicals forms a covalent linkage which connects the two polysaccharide chains. Thus, the structure of the dimers formed during gelation will depend on the radical position [48] (Figure 2(a)).

During gelling, the coupling of FA results in different structures. In AX gels, five di-FA, 8-5′, 8-O-4′, 5-5′, 8-5′ benzo, and 8-8′, have been detected, with 8-5′ and 8-O-4′ being the most abundant [48], and one tri-FA (4-O-8′/5′-5′) [84]. In addition, the presence of noncovalent weak interactions (hydrogen bonds) may contribute to the stability of the gel [85, 86].

During the formation of the AX gel, the FA is oxidized and disappears as a result of the formation of cross-links (di-FA and tri-FA). Nevertheless, the concentrations of di-FA and tri-FA formed at the end of gelation do not compensate for the decrease in the FA monomers. Therefore, the formation of superior FA oligomers (FA tetramers, FA pentamers) have been proposed by several authors [84, 86]. In the cell wall of cereals, five tri-FA, 5-5′/8-O-4′, 8-O-4/8-O-4′, 8-8′ (cyclic)/8-O-4′, 8-O-4′/8-5′ (noncyclic), 5-5/8-O-4′(H_2_O), and two FA tetramers, 4-O-8′/5–5′/8-O-4′ and 4-O-8′/5–5′/8–5′, have been identified and characterized [87]. The latest evidence suggests the possibility that the missing FA at the end of the gelling process could be related with the formation of superior structures that are not yet identified.

The AX gels present interesting features with a wide range of applications. These gels have neutral flavor, odor, and color as well as high water absorption and exhibit pH, temperature, and ionic stability [7]. They usually form quickly, and they are strong and thermostable [88]. In addition, they acquire a meso- and macroporous structure and have a dietary fiber nature. Due to their interesting characteristics, AX gels could be good candidates for their use as controlled release matrices for bioactive agents in the pharmaceutical, cosmetic, and food industries [7].

As previously mentioned, the antioxidant and prebiotic properties depend on the structure of AX. The presence of phenolic acids in AX, particularly FA, has been related to its prebiotic as well as antioxidant capacities. In addition, the resulting products of the enzymatic hydrolysis of AX exert important prebiotic properties. Then, it can be possible that the cross-linking process and formation of superior ferulate structures may contribute to some extent to the antioxidant and prebiotic properties of AX gels.

### 2.4. Preclinical Studies

Several *in vivo* studies have been performed in order to explore the potential of AX to exert its prebiotic, antioxidant, and anticancer effects. Following, the characteristics and findings of some of the most recent studies evaluating the effects of AX administration on animal models (mainly rats and mice) are presented.

#### 2.4.1. Prebiotic Effect

The prebiotic effect of AX and their derivatives, xylooligosaccharides (XOS) and AXOS, has been tested *in vivo*. Several studies using animal models (mainly rats) demonstrate the potential prebiotic effect of AX through different observations: showing a bifidogenic effect, modulation of mucosa and gut microbiota, and increasing SCFA levels (mainly propionic and butyric acid), among others.  Table 2 shows the characteristics and observations of some studies performed during the last 10 years related to the evaluation of the prebiotic properties of AX.

The supplementation of wheat AX to high-fat (HF) diet in mice led to an increase in bifidobacteria, particularly *B. animalis lactis*. In addition, AX modulated the microbiota by restoring the levels of bacteria (*Bacteroides-Prevotella* spp., *Roseburia* spp.) that were decreased with the HF. The bifidogenic effect of AX was correlated with lower levels of inflammatory markers in the serum which resulted in the improvement of the gut barrier functions. The consumption of AX also decreased body weight gain and fat mass development in the HF diet-induced obesity group. Moreover, the hypercholesteremia and the content of free cholesterol in the liver decreased in HF diet feeding. The presence of smaller adipocytes in the group treated with HF diet and AX was also observed which was attributed to the ability of AX to decrease the expression of genes involved in adipocyte differentiation, fatty acid uptake and oxidation, lipolysis, and fatty acid synthesis. The authors proposed a positive correlation between the modulation of microbiota and the antiobesity action as well as the cholesterol-lowering effect observed in the experiment [39]. Similar effects were observed when diet-induced obese mice were fed with a HF diet supplemented with wheat-derived AXOS. A positive correlation between the increase of bifidobacteria and improvement of metabolic endotoxemia and inflammatory markers was also observed with administration of AXOS. A higher expression of the tight junction proteins Z01 and claudin 3 led to a better function of the gut barrier. Moreover, the peptides GLP-1 and PYY, which are involved in the regulation of food intake and glucose homeostasis, respectively, increased after AXOS supplementation. This increase could be related with lower food intake and the improvement of insulin sensitivity in mice [13].

The supplementation of long-chain AX (LC-AX) in rats inoculated with human faecal microbiota has been associated with the production of propionic acid and stimulation of *B. longum*. LC-AX administration also increased the production of mucin, while it shifted mucin degradation from the caecum to the colon. The degradation of mucin in the distal regions of colon could be beneficial as most chronic colonic diseases, such as ulcerative colitis and colorectal cancer, originate in this region [90].

#### 2.4.2. Antioxidant Effect

The antioxidant property of AX has been related with several beneficial effects. *In vivo* studies, using rats, show that AX exerts its antioxidant effect by modulating lipid peroxidation, improving the activity of antioxidant enzymes, and protecting against oxidative stress. These positive effects have been related to the mechanisms of AX to exert its anticancer effect as well as improve lipid metabolic disorder and suppress lipid peroxidation.

Recently, male Sprague-Dawley fed with a HF diet supplemented with AX (HF-AX) showed lower triglyceride concentration in serum in comparison with the HF diet group. Higher lipoprotein lipase (LPL), hepatic lipase (HL), total lipase, and acyl-CoA oxidase (ACO) activities and lower triglyceride and cholesterol levels in the liver of the HF-AX group were observed. The authors suggest that intake of AX helped to alleviate lipid metabolic disorder by reducing triglycerides and low-density lipoprotein in serum of rats. The administration of HF-AX changed the lipid metabolism by improving the activity of fatty acid oxidation enzymes (LPL, HL, and ACO) which helped to reduce the triglyceride levels in liver. AX could help to maintain normal fat levels by activating lipid catabolism and oxidation rather than inhibiting lipid synthesis. Moreover, the activity of antioxidant enzymes glutathione peroxidase and total superoxide dismutase was also improved by the ingestion of AX resulting in a reduction of the oxidative stress in serum and tissues. The results also indicated that AX may alleviate the damage of hepatic morphology by regulation of liver cell apoptosis (Bax). These findings showed that supplementation of AX improved lipid metabolic disorder and alleviated liver damage by activation of liver lipid catabolism and suppression of lipid peroxidation in rats [92].

#### 2.4.3. Anticancer Effect

AX and AXOS have been investigated in order to explore their anticancer effects. The anticancer property of these polysaccharides on different types of cancer such as colon cancer, glandular stomach cancer, neuroblastoma, and liver cancer, among others, has been tested *in vivo*. According to the observations of the research done in the last 10 years, it is proposed that AX and AXOS may exert its anticancer effect through different mechanisms involving antioxidant, prebiotic, and immunomodulatory properties (Table 3).

The inhibition of growth of tumors in S180 tumor-bearing mice was significant when animals were administered with wheat bran AX. According to the results obtained in this study, it is suggested that AX exerts its antitumor activity via the improvement in immune response. The administration of AX enhanced the macrophage phagocytosis of chicken red blood cells (CRBCs) in tumor-bearing mice. The killing activity of NK cells from splenocytes in mice was increased, suggesting that AX could enhance the cytotoxic activity against spontaneously derived tumor cells. Moreover, AX treatment improved the production of IL-2 in blood serum of mice and subsequent proliferation of T cells, B cells, NK cells, and monocytes, increasing the cytotoxicity of T and NK cells. It is also proposed that the immunostimulating activity of AX may be related to the metabolism *in vivo* [16].

More recently, the chemopreventive activity of MGN-3/Biobran on glandular chemical induction of stomach carcinogenesis in rats was evaluated. The Biobran administration (40 mg/kg weight, 8 months) showed a significant reduction in the incidence of animals bearing dysplasia and adenocarcinoma. In addition, Biobran induced cancer cell apoptosis via cell cycle arrest of gastric cancer cells in the sub-G1 phase and also via the mitochondria-dependent pathway as indicated by upregulation of p53, Bax expression, downregulation of Blc-2, and an increase in Bax/Bcl-2 ratio. The authors suggest that the immunomodulatory effects of Biobran may represent another mechanism by which this agent suppresses the growth of adenocarcinoma [15].

### 2.5. Mechanisms Underlying the Anticancer and Antioxidant Effects of AX

#### 2.5.1. Immune-Modulation

In the last years, research efforts have focused on elucidating the mechanisms by which AX exerts its anticancer effects. In this regard, several studies have demonstrated that one of such mechanisms could involve the immune-modulation properties of AX.

**Figure 3:**
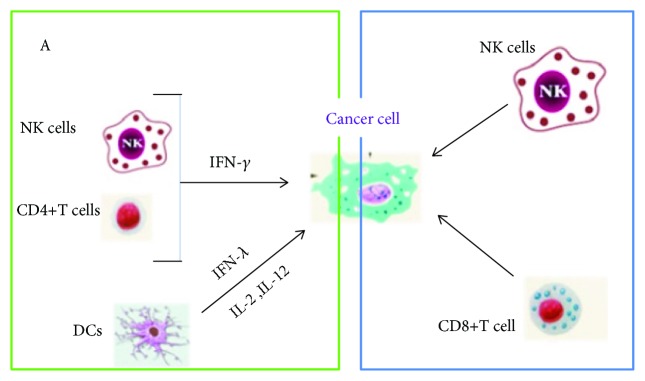
MGN-3/Biobran enhances the cytotoxicity reactivity of immune cells with anticancer effect and the production of certain cytokines (adapted from [41]).

It has been proposed that MGN-3/Biobran (AX from rice) exhibits its anticancer effects due to its ability to act as a biological response modifier (BRM). BRM are designed to activate the host immune response to destroy cancer cells [41]. Thus, Biobran has demonstrated to improve the activities of different arms of the immune system to attack cancer cells (Figure 3). Biobran improves the reactivity of cells with anticancer activity such as NK cells and CD8+ T cells and modulates the production of certain cytokines such as interferon-gamma (IFN-*γ*), -lambda (IFN-*λ*), IL-2, and IL-12 [41]. The basis of the mechanism behind the immune modulatory effects of Biobran is not completely elucidated. However, it is proposed that these AX are hydrolyzed in order to reduce its MW so they can diffuse through intestinal walls or directly into the blood stream and then be transported to the lymph nodes where immune cells reside [95, 96].

NK cells play an important role in the natural defense of the immune system against cancer and viral infections [93]. These cells work by attaching to cancer cells and releasing their granules which form holes causing cell death [41]. AX has the ability to increase the cytotoxicity activity of NK cells as confirmed by several *in vitro* and *in vivo* studies [16, 93]. Dendritic cells (DCs) are important antigen-presenting cells (APCs) involved in generating antitumor immune response. Biobran upregulates CD80 and CD86 which are molecules expressed on mature DCs. This stimulation promotes the production of proinflammatory and immune-regulatory cytokines. It is proposed that Biobran could bind to the cell surface receptors (TLRs and/or C type lectins) or to intracellular receptors (NLRP3 inflammasome) and trigger signaling pathways involved in cell activation and cytokine production [41].

A recent study evaluated the potential capacity of Biobran to activate and improve the cytotoxicity of NK cell activity against neuroblastoma *in vitro*, using several pediatric cell lines (acute leukaemia, neuroblastoma, Ewig sarcoma, embryonic rhabdomyosarcoma, and alveolar rhabdomyosarcoma) and *in vivo* (NOD/scid/IL-2R*γ*null neuroblastoma model) [93]. The stimulation of NK cells with Biobran resulted in a higher expression of the activation-associated receptors CD25 and CD69 than in unstimulated cells. In addition, the stimulation increased NK cell cytotoxicity against cancer cell lines and reduced the neuroblastoma growth *in vivo*. Several mechanisms are proposed in order to explain how Biobran could stimulate NK cell activity. One of those theories establishes an apoptotic effect mediated by the activation of NK cells releasing TNF-*α* and IFN-*γ* [75]. Other mechanisms could be related with the increasing of activating receptors on Biobran-stimulated NK cells. The authors observed an increase in the activation-associated receptor CD69 and CD25 on stimulated NK cells. The increase of CD69 is associated with a higher cytotoxicity of NK cells, while CD25 expression on NK cells is indicative of proliferation potential [97, 98]. NK cells attack cancer and viral cells through the release of their granules that cause cell death. In this regard, Biobran treatment increases the granular content (perforin and granzyme) of NK cells favoring its activity against malignant cells [99]. In addition, the treatment with Biobran helps NK cells to attach cancer cells [41].

AX can stimulate the production of interleukins such as cytokines IL-2 and IL-12 which are the main anticancer cytokines in humans [41]. In S180 tumor-bearing mice, the treatment with AX led to an increase in the secretion of IL-2 in the blood of mice. It is postulated that increase of IL-2 may be a mechanism for AX to exert antitumor effects, as IL-2 can improve the proliferation of T cells, B cells, NK cells, and monocytes and increase the cytotoxicity of T cells and NK cells [16, 100]. In addition, the ingestion of Biobran increased the production of IL-12 in multiple myeloma patients at one and two months post-ingestion [101].

AX can induce the production of TNFs (IFN-*λ* and IFN-*γ*) which have been found to exhibit antitumor activity [75, 101, 102]. In a previous study, the oral administration of partially hydrolyzed AX from corn husk to mice increased the production of IL-2 and IFN-*γ* and slightly increased IL-4 in mitogen-induced proliferation spleen cells. In addition, an increase in the activity of NK cells in spleen cells from transplanted-tumor mice was observed [102]. T helper 1 (Th1) promotes antitumor immunity through the production of IL-2 and IFN-*γ*, which activates NK cells to attack cancer and virus-infected cells [103]. In this study, the administration of AX also decreased ear inflammation of a model mouse of atopic dermatitis. According to the results obtained, it is suggested that the anti-inflammatory effect of AX is attributed to the activation of an IFN-*γ*-dependent Th1-like immune response in mice. In another study, multiple myeloma patients presented an increase of IFN-*γ* at two months post-treatment with MGN-3 [101].

T regulatory lymphocytes (T reg) or CD4+CD25+ lymphocytes act by suppressing the antitumor cytotoxic immune response [104]. In this regard, it is proposed that the counteracting of T reg cell activity could interfere in a positive way with the progression of neoplastic diseases by improving the efficacy of the anticancer immune response [41]. AX from rice was given orally to 22 patients with solid tumor for two months. The results showed an increase in TH cells, while T reg cells decreased, but the differences were not statistically significant. On the contrary, the TH/T reg ratio significantly enhanced after AX therapy [40].

#### 2.5.2. Induction of Apoptosis

Induction of apoptosis appears to be another mechanism by which MGN-3/Biobran may exert its anticancer effects. *In vivo* studies evaluated the effect of Biobran on mice bearing a SEC tumor as well as in chemically induced glandular stomach adenocarcinoma rats. According to the results observed in those experiments, the anticancer activity exhibited by Biobran was explained through a mechanism via induction of apoptosis.

The administration of Biobran in mice bearing a SEC tumor caused a significant delay in the volume and weight of the tumor in comparison to the control. The authors proposed that the antitumor activity of Biobran was related to its ability to induce apoptosis and immune modulation. The intraperitoneal treatment of Biobran on mice increased the number of apoptotic SEC cells. Moreover, cytokine production was influenced by increasing the levels of tumor necrosis factor-*α* and IFN-*γ*, while a downregulation of the immune suppressing cytokine IL-10 was observed. In addition, a considerable increase in NK cell activity was observed in mice treated with Biobran [75]. Biobran can cause tumor regression by the induction of cancer cell apoptosis via its immunumodulatory effects on NK cells and cytokine production. It is known that NK cells kill cancer cells by different pathways, and one of those involves the ligation of FasL to its Fas receptor to induce apoptosis [105]. It is also possible that Biobran could exert its apoptotic effect via the increase of TNF-*α* and IFN-*γ*. In this regard, both TNF-*α* and IFN-*γ* have been shown to act synergistically to induce cancer cell death through apoptotic and necrotic effects [106, 107].

Recently, the chemopreventive activity of Biobran against chemical induction of glandular stomach carcinogenesis in rats was associated with the ability of Biobran to induce apoptosis via the mitochondrial-dependent pathway in gastric cancer cells [15]. Biobran treatment caused a significant reduction in the incidence of animals bearing gastric dysplasia and adenocarcinoma in comparison to the untreated group. The upregulation in p53 expression, Bax expression, downregulation in Bcl-2 expression, the increase in Bax/Blc-2 ratio, and the activation of caspase-3 as well as the induction of cell-cycle arrest in the sub-G1 phase may explain the mitochondria-dependent pathway as the mechanism involved in the anticancer effect observed in the present study. Changes in p53, Bax, and Bcl-2 can alter the outer mitochondrial membrane and subsequent release of cytochrome C, which finally activates caspase-3. In this regard, Biobran has shown to induce apoptosis via activation of caspase-8, -9, and -3 [108]. Although the mechanism by which Biobran exerts its apoptotic effect is not completely elucidated, it could be related to the capacity of Biobran to sensitize the surface CD95 receptor that is involved in the triggering of apoptosis [109]. On the other hand, another possible mechanism by which Biobran suppresses the growth of tumor could be related to its immunomodulatory properties. It was observed that treatment with Biobran protected against chemical-induced lymphocytopenia in rats. Lymphocytes are white blood cells which are part of the immune system [15].

#### 2.5.3. Antioxidant

AX has been found to exert antioxidant effects through the modulation of lipid peroxidation, promoting the antioxidant defense system and protecting against oxidative stress [19]. Although the mechanisms behind the antioxidant property of AX are not fully understood, some studies suggest that its capacity to increase the activity of endogenous antioxidant enzymes, suppress lipid peroxidation, and induce apoptosis could be involved in such mechanisms [19, 92]. The antioxidant activity of AX has been related to its capacity to exert anticancer effect, improve lipid metabolic disorder, and alleviate liver damage in rats.

Noaman et al. [19] evaluated the antioxidant activity as a possible mechanism of Biobran to exert its antitumor potential on mice inoculated with Erlich ascites carcinoma (EAC) cells. Biobran administration suppressed tumor growth by normalizing the lipid peroxidation level and augmentation of glutathione (GSH) contents. In addition, the expression and activity of endogenous antioxidant scavenging enzymes (superoxide dismutase, glutathione peroxidase, catalase, and glutathione-S-transferase) in the cells of normal and tumor-bearing animals were increased in blood, liver, and tumor tissue. The ability of Biobran to induce apoptosis was proposed as the mechanism by which it could exert those antioxidant effects. Reactive oxygen species (ROS) act as signaling molecules for the initiation and execution of apoptosis. GSH and thioredoxin not only regulate ROS levels but could act as reversible redox modifiers of enzyme function [110]. In this regard, higher levels of GSH content in tissues of mice treated with Biobran were observed in comparison to the untreated mice. Among other functions, glutathione-S-transferase enzymes detoxify carcinogens [111]. This also could be a possible mechanism for Biobran to prevent cancer as elevated levels of such enzymes were observed in mice treated with Biobran.

The antioxidant capacity of AX has been also related to the improvement of metabolic disorder and alleviate liver damage in rats induced by high-fat diet [92]. Lipid peroxidation is one of the most common free radical chain reactions that causes oxidative damage [112]. In this study, mice fed with a high-fat diet presented an increase in serum and tissue oxidative stress and subsequent reduction of the antioxidant enzymes glutathione peroxidase and total superoxide dismutase as well as an increase in malondialdehyde level. On the contrary, supplementation of AX in high-fat diet catalyzed the dismutation of the superoxide (O_−2_) radical into either molecular oxygen (O_2_) or hydrogen peroxide (H_2_O_2_) by the promotion of total superoxide dismutase. In addition, the increase in glutathione peroxidase activity reduced lipid hydroperoxides [92]. On the other hand, AX supplementation could alleviate damage liver morphology by regulating the liver cell apoptosis through the modulation of the expression of proapoptotic and antiapoptotic proteins, Bax and Bcl-2. Moreover, Biobran has the ability to change the lipid metabolism by regulating the expression of UCP2, a mitochondrial membrane protein that works by accelerating the fatty acid *β*-oxidation and minimizing the production of reactive oxygen species [113]. These results may suggest that Biobran improved lipid catabolism and protected liver damage via an antioxidant mechanism.

## 3. Arabinoxylan Gels

### 3.1. Structural Parameters

**Figure 4:**
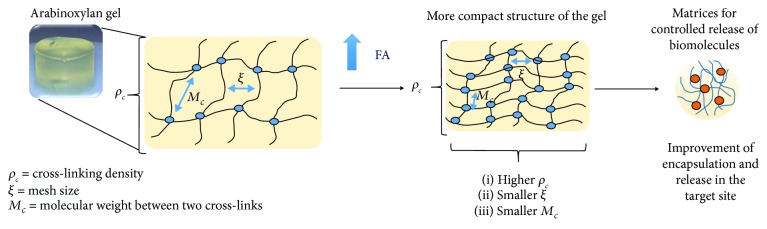
Structural parameters of ferulated AX gels.

The structural characteristics of the AX gels can be measured as a function of the determination of different parameters. The cross-linking density (*ρ*_*c*_), the mesh size (*ξ*), and the molecular weight between cross-links (*M*_c_) can be calculated by the determination of the swelling ratio of the gels [84] (Figure 4). These parameters allow to elucidate how the cross-linking between the AX chains could occur during the gel formation. Therefore, the knowledge of such structural characteristics is determinant to understand the gel properties and thus their possible applications.

The content of FA in AX is an important factor affecting the gel structure.  Table 4 shows the structural parameters and the FA content of maize and wheat AX gels at 2% (*w*/*v*). Usually, the increase in the FA content results in a decrease in mesh size and the molecular weight between cross-links, as well as an increase in the cross-linking density. This behavior results in the formation of gels with a more compact structure [9, 25, 84] (Figure 4). Gels with a compact structure are suitable for potential applications in the controlled release of biomolecules because they provide a better encapsulation and ensure the release of biomolecules in the site of interest.

The swelling ratio (*q*) indicates the amount of water that can be absorbed by the gel inside its tridimensional network. Martínez-López et al. [9] observed that when the AX concentration increased from 4 to 6% (*w*/*v*) in the gel, the *q* value decreased from 18 to 9 g water/g AX. Similar results have been reported by other authors [33, 84]. The results are explained in terms of a higher concentration of AX in the gel, which involves a high amount of FA, and it is related with a more compact polymeric structure that limits the water absorption capacity. In contrast, gels with lower polymer concentrations present higher *q* values due to a decrease in the covalent cross-links (di-FA and tri-FA). This decrease is attributed to longer uncross-linked chain sections in the gel, which facilitate its expansion, leading to high water absorption [114, 115].

The knowledge of the structural characteristics of the gel allows proposing different applications according to its functional properties. In this sense, we can take advantage about the quite relationship between the polysaccharide characteristics and the structural parameters of the gel (*ξ*, *M*_c_, and *ρ*_*c*_). This information can help to predict the gel structure and thus consider it for specific applications.

### 3.2. Microstructure

**Figure 5:**
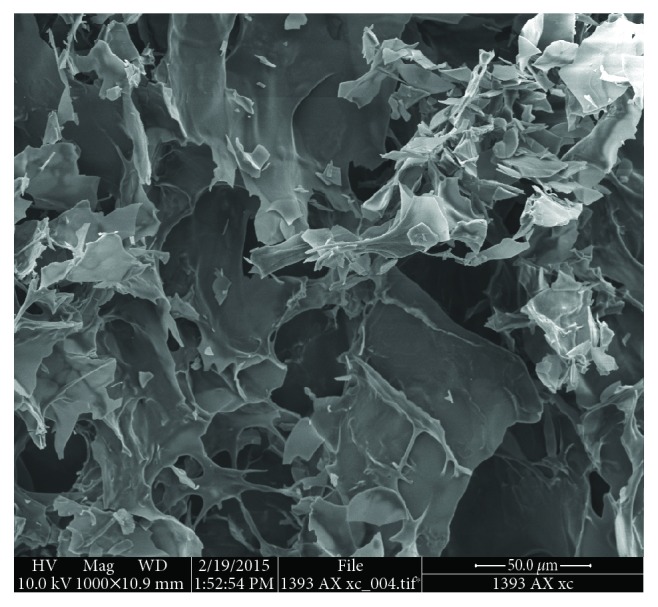
SEM image of the microstructure of lyophilized AX gel from maize at 1000x.

The microstructure of the AX gels has been studied by different microscopy techniques, with scanning electron microscopy (SEM) being the most used. The SEM micrographs have permitted a thorough understanding about the network of the AX gels. Several reports agree that lyophilized AX gels exhibit an imperfect honeycomb-like structure (Figure 5) [25, 35, 116]. This structure is mainly attributed to the polysaccharide characteristics. Although such structure could be established as a pattern for the gel networks, certain factors such as the molecule characteristics, structural parameters of the gel, or even the methods used prior to the analyses could affect the microstructure of these gels.

AX gels are characterized for presenting a porous and heterogeneous structure. However, depending on the polysaccharide characteristics, gels with different morphologies can be obtained. In SEM images, Martínez-López et al. [117] observed that maize bran AX gels showed an irregular honeycomb structure, while the nejayote AX gels appeared as a mix of sheets and rigid plates. The method used for freezing prior to the freeze drying process is another important factor affecting the gel microstructure. A rapid freezing (nitrogen immersion) of the gel results in the formation of smaller pores, compared to those obtained when using a slow freezing method [116, 118]. The freezing rate affects the quality of the frozen material, particularly those containing high amounts of water such as gels [118]. Therefore, a fast freezing results in a better-preserved structure of the gels.

Martínez-López et al. [119] evaluated the microstructure of AX microspheres by SEM. The microsphere's morphology presented a heterogeneous network, with irregular pore sizes and geometries, similar to those reported for other micro gels. In addition, the authors reported the presence of clusters of interconnected nodular structures. These clusters resulted in the formation of small pores (10–70 nm), while the binding between clusters led to a macroporous structure. The authors explain that the pore size of the gels formed via phenolic acid cross-linking is determined by the presence of nodular conglomerates.

The microstructural analysis of the gel provides a thorough understanding about the morphological characteristics of this material. Among other aspects, this analysis permits to study the conformation of the gel obtained as a result of the interactions between the polysaccharide chains. In this regard, the knowledge of the microstructural characteristics of the gel allows establishing a relationship between the morphology of the gel and its functional properties.

### 3.3. Viscoelastic Characteristics

The viscoelastic characteristics of AX can be studied by small-amplitude shear oscillatory rheology. The rheological analysis allows identifying the nature of a viscoelastic material, as well as the rheological behavior of the gel formed. The gelation ability of AX depends on the concentration of the polysaccharide, the Mw, the substitution degree, and particularly, the content of FA [26, 48].

**Figure 6:**
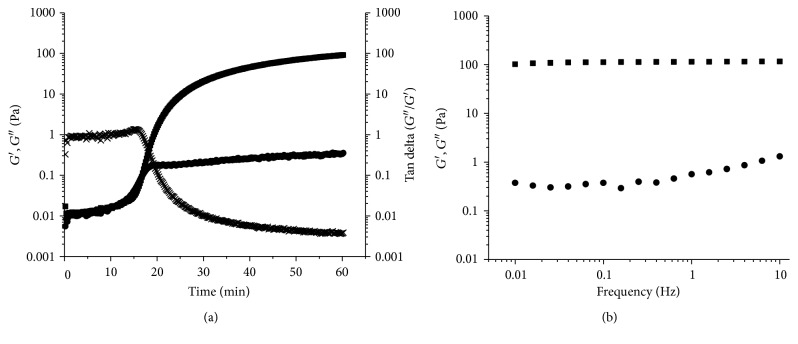
(a) Kinetic of gelation of AX solution (2% *w*/*v*) at 0.25 Hz and 5% strain (*G*′■, *G*^″^●, tan *δ* X) and (b) mechanical spectrum of the AX gel formed, registered at 5% strain (*G*′■, G^″^●).

The AX gels present a typical kinetic of a solid-like material. The kinetic of gelation of AX exhibits a rapid increase in the elastic modulus (*G*′), followed by a stability region, known as *plateau* [26] (Figure 6(a)). This behavior is due to the formation of covalent cross-links between the FA residues of the AX chains. The formation of cross-links in sufficient quantity limits the movement of the polysaccharide chains and leads to the formation of new cross-links. On the other hand, the mechanical spectrum for AX shows a behavior of a typical solid-like material with a linear *G*′-independent of time and a *G*^″^ much smaller than *G*′ and dependent of time [25, 84, 117] (Figure 6(b)).

Higher contents of FA result in the formation of stronger gels. Méndez-Encinas et al. [25] obtained stronger AX gels (*G*′ = 687 Pa) than Carvajal-Millan et al. [85] (*G*′ = 44 Pa), with a FA content of 6.05 and 2.3 *μ*g/mg AX, respectively, and using similar polysaccharide concentrations. The latter shows the impact of the FA content on the rheological properties of the gels.

### 3.4. Functional Properties

#### 3.4.1. Encapsulating

One of the most interesting properties of AX gels is their ability to encapsulate different agents. Several knowledge areas, such as the pharmaceutical, food, and medical, have special interest in investigating the encapsulating capacity of these gels. The research done on this respect ranges from the encapsulation of biomolecules and pharmaceuticals to cells (yeast and bacteria) [33, 37, 85, 86, 120]. The results have been quite promising, which has led to further research in this area.

In previous studies, Vansteenkiste et al. [86] evaluated the entrapment of a model protein (bovine serum albumin (BSA)) in AX gels and their protective effect against pepsin proteolysis and heating. The results indicated that the AX gel protected the embedded protein against enzymatic hydrolysis and also protected against heat.

Carvajal-Millan et al. [85] studied the capacity of AX gels with different FA contents to load model proteins. The results showed that the total protein loaded in the AX gels decreased as the concentration of AX in the gel increased. The authors proposed this decrease was due to the presence of a more compact network resulting from higher ferulate cross-linking structures, which turn down the protein movement within the gel. These results indicate that the structural characteristics of the gel affect its loading capacity, which in turn could also be true to proteins, other biomolecules, and even cells.

Regarding the medicine field, the search for, and the development of, novel encapsulating agents for the entrapment of probiotics has been of growing interest. Morales-Ortega et al. [37] evaluated the entrapment of bacteria (*B. longum* and *B. adolescentis*) in AX gels. The study showed the bacterial cells entrapped inside the network of the AX gel, suggesting that AX gels can be potential candidates for use in the entrapment of probiotics and even other cells of interest.

The encapsulation of small molecules such as methyl xanthine (caffeine) has also been investigated, with excellent results. Iravani et al. [35] encapsulated caffeine in AX gels. In addition, these gels were subjected to acidic conditions (HCl 0.1 M) in order to simulate the gastrointestinal fluid. AX gels are stable to acidic conditions due to its dietary fiber nature. Therefore, they have been a focus of study for their application as colon-targeted controlled-release systems.

#### 3.4.2. Colon-Targeted Drug Delivery Systems

Polysaccharide-based matrices have been widely studied for their use as colon-targeted drug delivery systems. Most of the time, the use of a single polysaccharide does not permit a targeted release, so it is common to use the combining and modifying of polysaccharides. The variations in the pH and transit time in the gastrointestinal tract result in a premature or even absent drug release [24]. AX can form covalent gels, which are stable to temperature [88], pH, and ionic strength changes [7]. Thus, AX gels have been considered as excellent candidates for their potential application in colon-targeted drug delivery systems.

AX gels have shown ability for use in controlled release of biomolecules, as well as cells (Table 5) [33, 35, 36, 85]. Previous studies evaluated the controlled-release properties of AX gels using model proteins such as ovalbumin, *β*-lactoglobulin, and insulin [33, 36]. The study of Berlanga-Reyes et al. [33] showed that only 11–18% of the proteins (insulin and *β*-lactoglobulin, resp.) was released by the end of a 15 h *in vitro* test. The authors suggest that AX gels could be used as a carrier to protect the proteins from the gastric environment and assure their release at the colonic region where AX gels can be fermented by the microbiota.

Polysaccharide hydrogels are a good alternative for the protection and controlled release of proteins and peptides in the colon. These systems protect biomolecules from protease degradation in the gastrointestinal tract and assure its release in the target site. Chitosan and alginate are the most studied polysaccharides for the controlled release of molecules. Although chitosan has been widely used in drug delivery, its solubility in acidic conditions (<pH 6.0) limits its application in the intestinal tract (>pH 6.5) due to the early degradation and release of the molecule [122]. Covalent AX gels resist changes in pH [27], allowing their stability in the acidic conditions of the stomach and subsequent degradation in the colonic region by the microbiota [32].

Paz-Samaniego et al. [32] designed core-shell AX particles for the entrapment of insulin and probiotics (*Bifidobacterium*). The authors evaluated the degradation and release using a simulator of the gastrointestinal tract (Simgi). The encapsulation efficiency was 72 and 90% for insulin and probiotic, respectively. The results showed that only 24% of the insulin was lost before the particles reach the colon, while 76% of the protein was released in the colon region, mainly in the transverse section. In addition, an increase in the *Bifidobacterium* population was observed due to the fermentation of the particles by these bacteria in the colonic region. This study suggests that AX particles could be excellent candidates for the controlled release of insulin and probiotics in the colonic region as an alternative for the treatment of diabetes. A low percentage of insulin release (33%) has been reported for insulin-loaded chitosan gel nanoparticles in pH 6.8 phosphate buffer solution (intestinal conditions). Moreover, *in vivo* studies showed a low hypoglycemic effect in diabetes-induced rats administered with the nanoparticles indicating the poor drug release in the colon. These results were attributed to the insolubility of chitosan in neutral and alkaline media, affecting the degradation and subsequent release of insulin in the colonic region. Concerning the encapsulation efficiency, the authors reported an efficiency of 84% which was a higher value in comparison to that observed in the AX microspheres [32, 122]. These differences could be related to the electrostatic charges between insulin and chitosan which favored its association.

Calcium alginate gels are also widely used for the controlled release of biomolecules. Insulin-loaded alginate microspheres present good encapsulation efficiency from 65 to 79% [123] which is similar to that reported in AX particles (72%) [32]. However, high percentage of insulin release (above 75%) has been observed under acidic conditions (pH 1.2) [123], resulting in a poor release in the intestinal region. This behavior is a consequence of dissociation of ionic linkages which leads to a weaker gel and release of the molecule by diffusion [123]. Lower release percentages of molecules (10–24%) have been observed using AX gels [32, 35] which could assure the release of a higher content of the molecule in the target site.

Swelling behavior is an important characteristic of hydrogels for its application as controlled-release systems. Hydrophilic groups of the polymer network lead to water uptake, resulting in a swelled gel that favors the release of the entrapped molecule by diffusion [124]. However, this also could be a disadvantage when gels exhibit high degrees of swelling because of the complete release of the molecule before reaching its target site. Swelling of alginate gels depends on the pH medium as they are sensible to ionic strength changes. In acidic conditions (pH 1.2), alginate gels have shown low swelling degrees, while in alkaline medium (pH 6.8–7.4) the swelling degree increases and then decreases abruptly because of the complete disintegration of the gel. In alkaline conditions, an ion exchange occurs between monovalent and divalent ions, resulting in the breakup of the gel network favoring swelling and subsequent degradation [124]. On the other hand, covalent AX gels resist pH changes so its swelling behavior is not affected by ionic charges. Among other factors, the swelling degree of AX depends on the content of FA as higher values of FA form higher content of cross-links leading to a more compact structure. The more compact the structure of the gel network, the less the capability of the gel to absorb water [25]. Thus, the swelling behavior of gels affects directly in drug release. In this regard, the percentage of drug (celecoxib) released from alginate gels in acidic medium was near 25%, while in alkaline conditions a complete release was observed at 8 h [124]. Similar values were reported for AX gels where 24% of insulin was released in stomach conditions, while 75% was released in the colon [32].

Iravani et al. [35] encapsulated methyl xanthine in AX gels and evaluated its release under acidic pH (HCl 0.1 M) in order to predict the behavior of the gels in the gastrointestinal tract. The results indicated that only 10% of caffeine is released at the beginning of a 3 h test. This suggests that a minimum of active molecules would be released in the stomach, while the rest of the drug could be dissolved later in the colon.

Recently, studies performed *in vivo* confirmed that insulin encapsulated in AX gels can be released into the colon [34]. In this study, insulin encapsulated in AX gels was administered to diabetic rats. The results showed a decrease in blood glucose levels, which suggest that insulin was released and absorbed in the colon of rats and maintains its functionality. The above results confirm that AX gels can transport the hormone through the gastrointestinal tract to the colon and to release it at this point, so they could be used as carriers for colon-specific drug delivery.

#### 3.4.3. Prebiotics

AX can be degraded by the colonic microbiota; however, the gelling process involves the cross-linking of the polysaccharide. Therefore, when discussing about the use of AX gels as colon-specific drug delivery systems, it is important to understand the impact of the gelling process on its degradation by the colonic microbiota. In this regard, an efficient release of the administered drug in the target site will depend on the capacity of the intestinal microbiota to ferment the gels.

**Figure 7:**
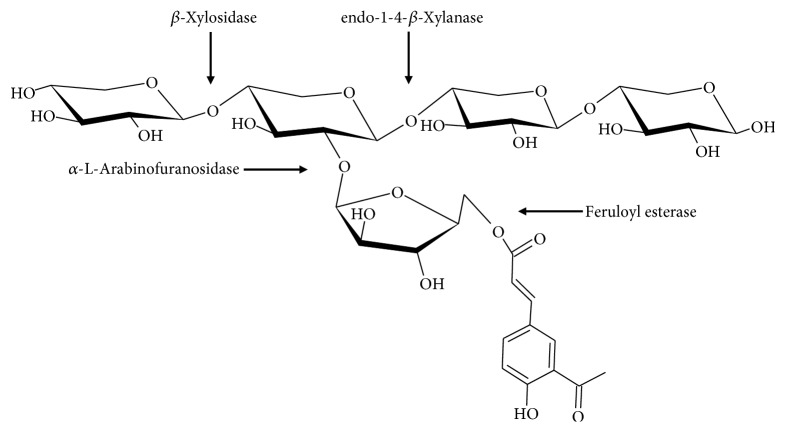
Sites of action of enzymes involved in AX degradation.

A combination of several specific enzymes is necessary for the complete degradation of AX (Figure 7). Among the main enzymes participating in its degradation are endo-1-4-*β*-xylanases (commonly called xylanases) which cleave the xylose backbone randomly to shorter fragments and *β*-xylosidases that attack the nonreducing ends of the xylose chain [125]. Other enzymes as *α*-L-arabinofuranosidades detach the arabinose residues from the xylan backbone, while FA esterases are needed to release the FA residues from AX [12]. The combination of these enzymes results in the transformation of AX to shorter fragments known as AXOS [12]. AX and AXOS degradation exhibits a bifidogenic effect by the increase of the *Bifidobacterium* population [12, 39].

The cross-linking of AX reduces the fermentation rate in comparison with the non-cross-linked molecule. The cross-links limit the access of bacterial enzymes for degrading the xylose chain. In addition, the cross-linking promotes a selective degradation, limiting the growth of *Bacteroides*. This bacterial genus does not produce the endoxylanases necessary to degrade the xylan chain, resulting in the incomplete degradation of the polysaccharide and therefore in a limited growth of these bacteria. In this regard, the selective degradation limits the growth of bacteria that are not considered beneficial at all, but it may favor the growth of bacteria that are so [10].

In a recent study, the ability of AX gels to modulate the gut microbiota and lipid metabolism in high-fat diet-induced obese rats was evaluated. The results showed that administration of a high-fat diet with AX gels increased the levels of *Bifidobacteria* and decreased those of *Bacteroides* in comparison with the high-fat diet and control groups. Moreover, the addition of AX to high-fat diet resulted in smaller adipocytes in comparison with the control and high-fat diet groups as was observed by histological analyses of the subcutaneous adipose tissue. These findings suggest the potential prebiotic properties of AX gels by the modulation of microbiota and antiobesity effect in high-fat diet-induced obese rats [126].

Recently, the degradation of AX gels at different concentrations (4 and 6% *w*/*v*) was compared. In this study, a slower degradation in the gels of higher concentration was observed. The authors explained this behavior was attributed to the higher cross-linking density of the gels, which results in a more compact structure and limits the access for the enzymes [9]. In this regard, it can be suggested that although a high cross-linking density decreases the fermentation rate of the AX gels, this can favor the growth of probiotic bacteria. Since the AX gels present potential prebiotic activity, they also could exhibit beneficial effects (possibly anticancer) due to the positive effects of their fermentation products (SCFA) and the increase of probiotic bacteria. Thus, the AX gel application as a colon-targeted drug delivery system would be highly possible, which would also exert effects with anticancer properties.

#### 3.4.4. Antioxidant

The antioxidant activity of the AX has been extensively related with their FA content. Nevertheless, only few studies have explored the effect of the oxidative gelation of AX regarding this property. Although the gelling of AX involves the oxidative coupling of FA, the cross-links could be affecting in some way the antioxidant activity of the AX.

It is known that the structural features of AX affect the fermentability of AX, and therefore, it will also impact its antioxidant capacity. The fermentation patterns of AX and cross-linked AX differs in how the bacterial enzymes can degrade the molecule. In AX, *α*-arabinofuranosidades begin with the detaching of the arabinosyl moieties and subsequent fermentation of the available xylose chain. On the contrary, in AX gels the arabinose moieties are firstly utilized and even when the side branches are removed, the arabinose residues are preferred over the xylan chain. This behavior is because the gel-like structure restricts the access of xylanolytic enzymes to their target sites and the xylan backbone becomes more resistant to fermentation [10]. In AXOS, the presence of FA esterified to arabinose results in a steric effect that limits the access of arabinofuranosidases to their target sites and also slows down the activity of FA esterases, leading to a decrease of the degradation and subsequent fermentation of the molecule [20].

It has been demonstrated that the presence of FA and di-FA in cereal brans can be released by FA esterases present in the gastrointestinal tract (intestinal mucosa and microbiota) of humans and rats [127]. In this way, probiotics present in the colonic microbiota, such as *Lactobacillus* and *Bifidobacterium*, are able to produce FA esterases, enzymes required for the release of the FA moieties from AX and AXOS [21, 55, 56]. A previous study showed that the presence of two *Bifidobacterium* strains (*B. longum and B. adolescentis*) is required for the fermentation of maize bran AX gels [9]. Therefore, even if some bacteria produce the specific enzymes to release the FA moieties in AX gels, the cross-feeding between different bacteria producing different enzymes is highly needed for the complete degradation of AX and AX gels.

The antioxidant capacity of phenolic acids depends on uptake and further metabolism [127]. Once the FA is released, it is then metabolized by the intestinal microbiota. The FA can be transformed into phenylpropionic acids and/or vinyl phenol derivatives, which still exert certain antioxidant capacity but in a lower extent than FA. According to this, it could be stated that the metabolism of FA by bacteria will lead to a decrease in its antioxidant capacity as has been demonstrated by Snelders et al. [20]. In this study, AXOS with FA esterified to arabinose exhibited good antioxidant capacity; however, the release and subsequent metabolization of FA decreased its antioxidant capacity. The authors suggested that AXOS-bound FA will reach the colon, where FA can be released and then metabolized by FA esterases located in this region [20]. In the colonic region, FA (bound or free) as well as its metabolites can exert its antioxidant activity through a direct interaction with the colon epithelium cells and thus reduce colorectal cancer [128]. On the other hand, the absorption of di-FA from cereal bran in the gut and its subsequent reach to the vascular system was demonstrated in rats [127]. These compounds also exhibit good antioxidant capacity and may exert its beneficial effects in the colonic region as they can interact directly with the intestinal barrier.

Previous studies have shown that not only the level of FA but also the condition under which it appears (free, bond, or dimerized) impacts the antioxidant capacity of AX [129]. The presence of dimers and trimers (di-FA, tri-FA) or more complex ferulated structures decreases the antioxidant capacity of AX in comparison to the FA molecule esterified to AX. The availability of hydroxyl groups and resonance systems of FA vary according to the cross-linking position and are important factors to exert the antioxidant function of FA [87]. Thus, it is confirmed that the gelling process impacts on the antioxidant capacity of the gels.

Several studies agree that the antioxidant activity of AX decreases after the gelation process due to the formation of di-FA and tri-FA [8, 119]. It is well documented that the formation of the AX gel involves the cross-linking of FA and consequently its oxidation. Nevertheless, the di-FA and tri-FA amounts produced during gelation do not compensate the FA oxidized. The remnant FA can continue to react and form higher ferulated structures [84]. Trimers and tetramers of FA have been identified in the cell wall of cereal grains, and even more complex ferulated structures are suggested [87]. Since the remaining FA could react and continue to form more complex structures, then it would be possible that such FA could keep stabilizing the free radicals and therefore exhibit antioxidant activity.

According to the above mentioned, the antioxidant activity of AX gels could be mainly related to the FA remaining after the gelation process. Thus, a higher FA content in AX gels may result in better antioxidant activity. AX with higher FA content (6 *μ*g/mg AX) can form gels with high cross-linking density and still exhibit antioxidant activity [8]. In addition, these gels preserve high amounts of FA after their gelation [130], which would allow them to exert a greater antioxidant activity.

The antioxidant activity of AX gels depends on the polysaccharide structure. The content and appearance of FA in the gel are the main factors affecting such property. AX gels with high cross-linking density can exhibit antioxidant activity. In this logical order, since the anticancer activity of AX is related to its antioxidant activity, the AX gels could also exhibit this property. However, the evaluation of the anticancer activity of AX gels and its antioxidant property relationship is necessary. These findings could allow obtaining AX gels for colon-targeted drug delivery systems with protective effect against colon cancer.

Ferulated AX exhibit prebiotic and antioxidant properties, which confer anticancer potential to the polysaccharide. Since AX gels preserve their prebiotic and antioxidant properties, they would also exert antiproliferative activity. Furthermore, the high cross-linking density of the gels would increase such properties. In recent years, the investigation of the properties of AX gels has been the focus of numerous studies due to its potential application as colon-targeted drug delivery systems. In this regard, it is necessary to evaluate the effect of gelation on the antiproliferative activity of AX. Moreover, the relationship between the structural characteristics and the functional properties of the AX gels should be investigated, particularly the effect of the high cross-linking density of the gels on their antiproliferative activity. High cross-linking density AX gels could be a good alternative for the colon-targeted drug delivery in the treatment of colon cancer.

## 4. Conclusion

This review describes the functional properties of AX and AX gels and their potential application as antioxidant and anticancer agents. Ferulated AX have prebiotic, antioxidant, and anticancer properties which can be exploited by the pharmaceutical field. Recently, the research has focused in the development of novel colon-targeted delivery systems for the treatment of colon associated diseases, such as inflammatory bowel disease, irritable bowel disease, and particularly colon cancer. Due to their gelling ability, AX have gained interest as promising polysaccharides for the designing of drug delivery systems with bioactive properties such as prebiotic, antioxidant, and anticancer.

The antioxidant and prebiotic activity of AX has been previously demonstrated. The fermentation products of AX exhibit a prebiotic effect, providing benefits for the gut health. Moreover, its antioxidant activity exerts a protective effect against the free radicals and oxidative stress. Regarding the gelling ability of AX, specific studies are needed to elucidate such beneficial effects in the AX gels. The anticancer activity of AX appears to be strongly related to its antioxidant and prebiotic properties. Future studies will be needed to evaluate the effect of gelation on the anticancer activity of AX gels. Furthermore, this information will allow establishing a relationship between the anticancer activity of AX with its antioxidant and prebiotic properties.

The AX gels are promising candidates for application as colon-targeted drug delivery systems for the treatment of colon cancer. However, only few studies regarding the evaluation of the antioxidant and prebiotic effect of AX gels have been conducted *in vitro*, and no evidence of *in vivo* data is available. Therefore, it is important to conduct research on the performance of *in vitro* and *in vivo* studies focusing on the effect of gelation on the biological properties (prebiotic, antioxidant, and anticancer) of AX and to elucidate the relationship between the properties of the gel by itself, not only as a mere carrier.

## Figures and Tables

**Table 1:** Origin, FA content, and current/potential application of AX in biomedical and pharmaceutical fields.

AX origin	FA content (*μ*g/mg AX)	Currently/potential application in biomedical/pharmaceutical field	Reference
Nejayote/maize bran	0.012/0.025	Entrapment of probiotic	[32]
Nejayote/maize bran	0.012/0.025	Entrapment and controlled release of insulin and probiotics targeted to colon	[11]
Maize bran	0.34	Controlled release of insulin and *β*-lactoglobulin	[33]
Maize bran	0.25	Controlled release of insulin targeted to colon	[34]
Maize bran	4.0	Entrapment and controlled release of methyl xanthine	[35]
Wheat endosperm	2.3	Controlled release of proteins	[36]
Wheat endosperm	0.53	Entrapment of probiotics	[37]
Wheat bran	0.435	Antitumor and immunomodulatory activity	[16, 38]
Wheat bran	—	Prebiotic	[39]
Rice bran	—	Antitumor and immunomodulatory activity	[40]
Rice bran (MGN-3/Biobran)	—	Cancer immunotherapy Prevention and inhibition of cancer Synergistic effect with chemotherapeutic agents	[15, 41, 42]
Finger millet bran	0.001	Immunomodulatory activity	[29]
Ispaghula (*Plantago ovata*) seed	nd	Drug carrier	[31, 43]
Ispaghula (*Plantago ovata*) seed	—	Controlled release of mucoadhesive oral films	[44, 45]

nd: no detectable; −: no reported.

**Table 2:** *In vivo* studies on the evaluation of the prebiotic effect of AX.

Animal model	Diet/experimental time	Findings	Reference
Male chickens	Control diet (CT), diet supplemented with XOS, wheat bran-derived AXOS, wheat endosperm alkali-solubilized AX. 2 w	All treatments increased bifidobacteria. AX decreased body weight gain after 2 weeks of feeding compared with CT.	[89]

Male C57bl6/J mice	Control diet, high-fat (HF) diet, HF diet supplemented with AX. 4 w	HF diet supplemented with AX restored microbiota with a major effect on *Roseburia* spp., *Bacteroides-Prevotella* spp., and bifidobacteria. Improvement of gut barrier function, decrease in adipocyte size, fatty acid uptake, fatty acid oxidation and inflammation, and decrease in key lipogenic enzyme activity in the subcutaneous adipose tissue.	[39]

Male germ-free Fisher 344 albino rats inoculated with human faecal microbiota	Control diet, diet supplemented with long-chain AX (LC-AX) and diet supplemented with inulin (IN). 6 w	LC-AX and IN increased SCFA levels (propionate and butyrate, resp.). Stimulation of butyrate-producing bacteria and bifidobacteria, respectively. Reduction of mucin-degrading *Akkermansia muciniphila* and more mucin production by the host. Less weight gain.	[90]

Male Wistar rats	Diets supplemented with WU-AX, WE-AX, and AXOS. 14 days	WU-AX supplementation increased butyrate production and butyrate-producing bacteria. WE-AX and/or AXOS reduced pH, suppressed relevant markers of proteolytic breakdown, and induced selective bifidogenic response. Combination of WU-AX, WE-AX, and AXOS showed a synergic effect.	[91]

Male C57bl6/J mice	Control diet, high-fat (HF) diet, HF diet supplemented with AXOS. 8 w	AXOS supplementation exerted a bifidogenic effect. Improvement of the HF-induced body weight gain, fat mass development, hyperinsulinemia, insulin resistance, endotoxemia, and inflammatory disorders in a model of HF diet-induced obesity.	[13]

Pigs	Low dietary fiber and high-fat diet (WSD), AX-rich diet (AXD), and resistant starch diet (RS). 3 w	AXD feeding shifted the microbial composition towards butyrogenic species in the faeces and increased the large-intestinal butyrate pool size.	[59]

w: week.

**Table 3:** Description of studies evaluating the anticancer potential of AX and AXOS, *in vivo*.

Type of cancer/animal model	Carcinogenic agent/cancer cells	Dosage/experimental time	Findings	Reference
Solid Erlich carcinoma Female albino mice	Erlich ascites, carcinoma cells, and intramuscular inoculation	MGN-3/Biobran (25 mg/kg bw) ip Six times/week for 25 days at either day 4 or day 11 post-cancer cell inoculation.	MGN-3 suppressed the growth of tumors, normalized lipid peroxidation, and increased glutathione contents. Increased activity of endogenous antioxidant scavenging enzymes (superoxide dismutase, glutathione peroxidase, catalase, and glutathione-S-transferase) in blood, liver, and tumor tissue.	[19]

Colon carcinogenesis Male F344 rats	1,2,-Dimethylhydrazine (DMH), subcutaneous injection.	High-fat diet plus AXOS (48 g/kg). 10 days before receiving carcinogen and continued for 13 weeks.	Lower counts of preneoplastic lesions (mucin depleted foci (MDF)) in comparison to the control group. Fewer preneoplastic lesions (aberrant crypt foci (ACF)) in the distal part of the colon.	[17]

S180 tumor-bearing mice ICR male mice	Mouse sarcoma S180 cells, intramuscular inoculation.	AX orally administered (100, 200, and 400 mg/kg bw).	Administration of AX significantly inhibited the growth of mouse transplantable tumors and promoted thymus and spleen indexes, splenocyte proliferation, NK cell and macrophage phagocytosis activity, and IL-2 production. Increased peripheral leukocyte count and bone marrow cellularity.	[16]

Neuroblastoma NOD-scid IL-2Rgnull mice	Injection of NB1691luc cells.	NK cells activated with 100 *μ*g/mL MGN-3/Biobran injected intravenously. 7 days after injection of tumor cells and performed twice a week for 4 weeks.	Significant inhibition of neuroblastoma growth and improvement in survival in the group treated with Biobran. Increase in the activation-associated receptors CD69 and CD25 on NK cells.	[93]

Glandular stomach carcinogenesis. Male Wistar rats	Methylnitrosoguanidine (MNNG), via oral gavage.	MNNG plus Biobran (40 mg/kg bw) every other day via oral gavage. 8 months	Biobran reduced incidence of animals bearing gastric dysplasia and adenocarcinoma. Decrease in expression of tumor marker Ki-67 and increase in the level of apoptotic gastric cancer cells via cell cycle arrest (sub-G1) and mitochondria-dependent pathway. Protection against lymphocytopenia.	[15]

Hepatocarcinogenesis. Male albino rats	N-nitrosodiethylamine (NDEA) and carbon tetrachloride (CCl_4_).	MGN-3/Biobran (25 mg/kg bw), 5 times/week ip 2 weeks prior to receiving carcinogen and continued for 20 weeks.	Reduction in liver tumor incidence, decrease of preneoplastic foci in hepatic parenchyma, and inhibition of development of hepatocellular carcinoma. Regulation of AST, ALT, ALP, and gamma GT levels. Increase in cell cycle sub-G0/G1 population. Downregulation of expression of NF-*κ*Bp65 and Bcl2, upregulated p53, Bax, and caspase-3 and increased the Bax/Bcl-2 ratio.	[94]

bw: body weight; ip: intraperitoneal; AST: serum aspartate aminotransferase; ALT: alanine aminotransferase; ALP: alkaline phosphatase; gamma GT: gamma glutamyl transpeptidase.

**Table 4:** Structural parameters of AX gels at 2% (*w*/*v*).

Origin of AX	*M*_c_ × 10^3^ (g/mol)	*ξ* (nm)	*ρ*_*c*_ × 10^−6^ (mol/cm^3^)
Wheat (2 *μ*g AF/mg AX)^a^	119	201	14
Maize (6 *μ*g AF/mg AX)^b^	34	96	67

^a^[85]. ^b^[25].

**Table 5:** Potential application of AX gels in controlled release of biomolecules and cells.

AX source	Biomolecule/cell	Potential controlled-release system	Reference
Wheat	Ovalbumin (Ov)	Ov-AX gels for entrapment and controlled release of proteins. 70–88% protein release as Ov/AX ratio increased (24 h *in vitro* test).	[36]
Maize bran	Insulin and *β*-lactoglobulin	AX gels for controlled release of proteins. 11–18% of protein release at the end of a 15 h *in vitro* test.	[33]
Maize bran	Lycopene	Lycopene/AX gels for controlled delivery of biomolecules. 3–4% lycopene release at the end of a 4 h *in vitro* test.	[121]
Maize bran	Methyl xanthine (caffeine)	AX microparticles. 10% of caffeine release at a 3 h *in vitro test* (0.1 M HCl).	[35]
Ispaghula (*Plantago ovata*) seed husk	Metronidazole hydrochloride (MH)	MH-loaded calcium gelled AX microspheres for extended drug delivery. 90% MH release at a 70–80 min *in vitro* test.	[43]
Maize bran	Insulin	AX microspheres as insulin carriers for colon-specific drug delivery. Insulin release in the colonic region of diabetic rats.	[34]
Maize waste water (nejayote) and maize bran	Insulin, *Bifidobacterium*	Core-shell AX particle (AX-insulin/AX-*Bifidobacterium*) for entrapment and delivery of insulin and probiotics targeted to colon for diabetes treatment. 76% insulin release in colonic region using a simulator of the gastrointestinal tract (Simgi).	[32]
